# The clinical effectiveness of the STUMBL score for the management of ED patients with blunt chest trauma compared to clinical evaluation alone

**DOI:** 10.1007/s11739-022-03001-0

**Published:** 2022-06-23

**Authors:** Elena Callisto, Giorgio Costantino, Andrew Tabner, Dean Kerslake, Matthew J. Reed

**Affiliations:** 1 Emergency Medicine Research Group, Department of Emergency Medicine, Royal Infirmary of Edinburgh, 51 Little France Crescent, Edinburgh, EH16 4SA UK; 2 Pronto Soccorso, ASST Lodi, Largo Donatori del Sangue 1, 26900 Lodi, Italy; 3 Pronto Soccorso e Medicina D’Urgenza, Fondazione IRRCS Ca’ Granda Ospedale Maggiore Policlinico di Milano, Via Francesco Sforza, 28, 20122 Milan, Italy; 4 REMEDY (Research Emergency Medicine Derby), University Hospitals of Derby and Burton NHS Foundation Trust, Derby, UK; 5 Department of Emergency Medicine, Royal Infirmary of Edinburgh, 51 Little France Crescent, Edinburgh, EH16 4SA UK; 6 Acute Care Edinburgh (ACE), Usher Institute of Population Health Sciences and Informatics, University of Edinburgh, Nine Edinburgh BioQuarter, 9 Little France Road, Edinburgh, EH16 4UX UK; 7 Università degli Studi di Milano, Facoltà di Medicina e Chirurgia, Via Festa del Perdono, 7, 20122 Milan, Italy

**Keywords:** Thoracic injuries, Rib fractures, Trauma, Score

## Abstract

The STUMBL (STUdy of the Management of BLunt chest wall trauma) score is a new prognostic score to assist ED (Emergency Department) decision making in the management of blunt chest trauma. This is a retrospective cohort chart review study conducted in a UK University Hospital ED seeing 120,000 patients a year, comparing its performance characteristics to ED clinician judgement. All blunt chest trauma patients that presented to our ED over a 6-month period were included. Patients were excluded if age < 18, if they had immediate life-threatening injury, required critical care admission for other injuries or in case of missing identification data. Primary endpoint was complication defined as any of lower respiratory tract infection, pulmonary consolidation, empyema, pneumothorax, haemothorax, splenic or hepatic injury and 30-day mortality. Clinician judgement (clinician decision to admit) and STUMBL score were compared using the receiver-operating curve (ROC) and sensitivity analysis. Three hundred and sixty-nine patients were included. ED clinicians admitted 95 of 369 patients. ED clinician decision to admit had a sensitivity of 83.9% and specificity of 86.0% for predicting complications. STUMBL score ≥ 11 had a sensitivity of 79.0% and specificity of 77.9% for the same and would have led to 117 of 369 patients being admitted. Area under the curve (AUC) of STUMBL score and ED clinician decision to admit was 0.84 (95% CI 0.78–0.90) and 0.85 (95% CI 0.79–0.91), respectively. Our findings show that a STUMBL score ≥ 11 performs no better than ED clinician judgement and leads to more patients being admitted to hospital.

## Introduction

Blunt chest trauma accounts for around 15% of all Emergency Department (ED) trauma presentations worldwide with significant morbidity and mortality [1–4]. Currently, no evidence-based guidelines exist to assist in the management of this patient group unless the patient has severe, immediate life-threatening injuries [1, 4–6]. Decisions around the ongoing management of non-life-threatening blunt chest wall trauma patients in the ED is difficult due to the frequent onset of delayed respiratory complications and clinical symptoms in the ED are not considered an accurate predictor of outcome [1, 2, 6–9].

Several scores have been proposed in the literature to help predict complications and guide management of non-major blunt chest trauma. However, most were designed and validated in patients with multiple injuries [1, 10, 11]. Battle et al. [1] have derived and validated a new prognostic risk score to predict complications and guide the management of blunt chest trauma patients but have not yet assessed the clinical impact of the score. As shown in Table 1, the STUMBL (STUdy of the Management of BLunt chest wall trauma) score (also referred to as the Battle score) includes five predictors: age at attendance, number of rib fractures, chronic lung disease, use of pre-injury anticoagulants and oxygen saturation (SpO_2_). This is the first score to introduce clinical variables, specifically chronic lung disease and anticoagulation, in contrast to other scores which have used anatomical variables and age alone [10, 12]. A huge benefit of the STUMBL score is that these variables are all routinely measured in the ED.

**Table 1:** The STUMBL score. Adapted from Battle et al. [1]

	Score	
Age	1 point for each decade: 10–19 scores 1, 20–29 scores 2, etc	
Number of rib fractures	3 points per rib fracture	
Pre-injury anticoagulants	No	0
	Yes	4
Chronic lung disease	No	0
	Yes	5
Oxygen saturation levels	100–95%	0
	94–90%	2
	89–85%	4
	84–80%	6
	79–75%	8
	74–70%	10
Risk score	Probability of developing complications as reported by Battle et al.	
0–10	13%	
11–15	29%	
16–20	52%	
21–25	70%	
26–30	80%	
31+	88%	

The score had a sensitivity of 80%, specificity of 96%, positive predictive value (PPV) of 93% and a negative predictive value (NPV) of 86% for predicting complications following blunt chest wall trauma. The authors suggested a score of 11 or greater as the cutoff point for a significant risk of developing complications suggesting hospital admission, and a score of 26 as the cutoff at which the patient was at sufficiently high risk to warrant critical care admission.

The aim of this study was to investigate the clinical effectiveness of the STUMBL score for the management of blunt chest trauma patients in the ED compared to clinical evaluation alone.

## Methods

### Study design and setting

This was a single-centre retrospective cohort study conducted in a UK University Hospital ED seeing 120,000 patients a year in Edinburgh, Scotland. The study was conducted over a 6-month period from the 1st January 2019 to 30th June 2019. The study was deemed an NHS Lothian service evaluation and, therefore, did not require formal Regional Ethics Committee review.

### Participants

We included all patients ≥ 18 years old with an ED diagnosis of blunt chest trauma. We excluded patients if they had sustained any immediate life-threatening injury (defined as physiological instability), if they required critical care admission (High Dependency Unit; HDU or Intensive Therapy Unit; ITU) for other injuries, or if identification data were missing.

### Data collection

An electronic search was carried out on our Electronic Patient Record (EPR) system (MedTRAK, Intersystems) to identify all patients with an ED diagnosis coded under the following criteria ‘Heading = Chest, Category = Trauma’ or ‘Heading = Ribs, Category = Fracture, Dislocation or Musculoskeletal’. This search was cross-referenced with all patients in the South East Scotland Scottish Trauma Audit Group (STAG) database with an Abbreviated Injury Scale (AIS) score > 0 in the ‘Thorax’ body region to ensure no patient were missed.

Researcher EC (unblinded) collected data retrospectively for each patient from the Electronic Patient Record (EPR) and Emergency Care Summary (ECS) medical record systems on a standard abstraction form which was later imported into Microsoft Excel for analysis. In Lothian, all patient attendance data is recorded electronically. We assigned number of rib fractures based on the formal radiology report of the best available imaging (chest radiograph; CXR or computed tomography; CT). If imaging was not performed then a score of 0 was assigned. When the exact number of rib fracture was not reported in the formal radiology report, this was assigned based on consensus imaging opinion by 2 independent examiners.

We collected oxygen saturation data based on the first room air (RA) oxygen saturation measurement in the ED. If RA SpO_2_ was not reported (39 patients; all discharged), then we assigned a normal value (i.e. 95–100%). If only SpO_2_ on oxygen was reported (12 patients), then we assigned a score based on this. If there was no record in the patient’s medical notes of chronic lung disease or use of pre-injury anticoagulants, then we assumed they were absent. We defined chronic lung disease as the presence of chronic active pulmonary disease such as chronic obstructive pulmonary disease (COPD). We did not include patients with a past medical history of asthma.

We also extracted the following data from electronic medical records: age, sex, mechanism of injury, associated injuries, comorbidities, respiratory rate (RR), presence or absence of flail chest, fracture involving any of first 4 ribs and presence or absence of sternal fracture. Researchers EC and MJR resolved any data queries by consensus following discussion.

### Primary endpoint

A complication was defined by documentation in the medical records of one or more of the following: clinical lower respiratory tract infection (LRTI) as per treating clinician decision, pulmonary consolidation on imaging (undifferentiated contusion or infection), empyema, pneumothorax (PNX), haemothorax, splenic or hepatic injury, and 30-day mortality.

### Statistical analysis

We used a standardised data abstraction form to collect all data and missing data were recorded as missing. We entered data into a specially designed Microsoft Excel (Microsoft Corporation, Redmond, Washington, USA) database for statistical analysis. Data are presented as median with interquartile range (IQR) (25th to 75th percentile) for non-parametric continuous variables and as simple frequencies, proportions and percentages for categorical variables. Parametric continuous variables are presented as mean with 95% confidence interval (CI). Clinician judgement, STUMBL score and complications are described and compared using the receiver-operating curve (ROC) and sensitivity analysis. Sensitivity, specificity, positive predictive value (PPV) and negative predictive value (NPV) were calculated using the two-by-two tables.

### Sample size

In the original derivation cohort, 161 of 274 (59%) patients had a complication. Using the one in ten rule, because the STUMBL score has 5 predictive variables, we would require 50 events to validate the rule. In the original STUMBL population with a 59% complication rate, this would equate to needing to study 85 patients. Because of the reduced complication rate in the original validation cohort (103 of 237; 43%), we chose to study at least twice this number (allowing for a reduced complication rate of 30%), and therefore, chose to study a 6-month period of ED presentations.

## Results

### Characteristics of study subjects

**Fig. 1:**
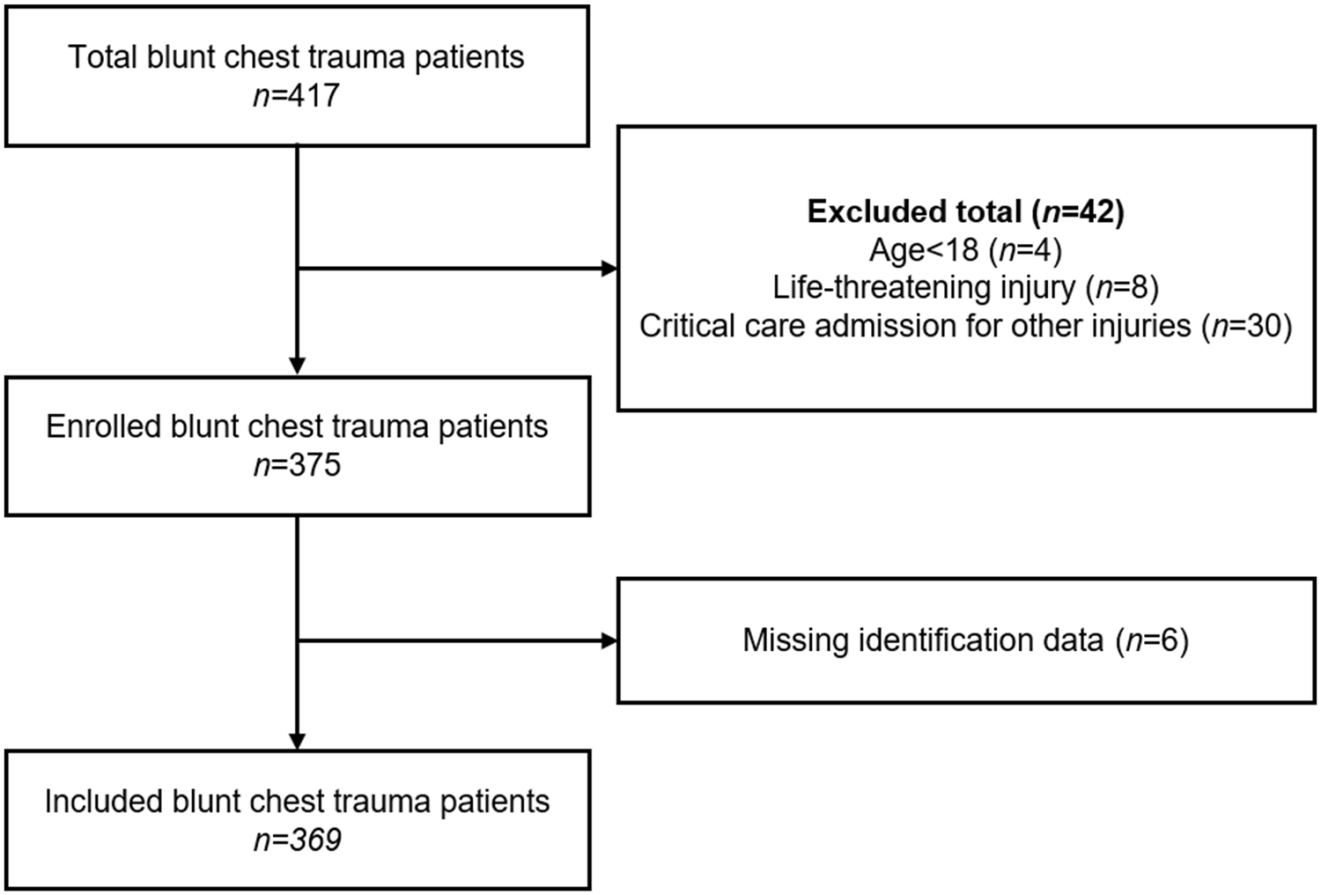
Diagram showing flow of patients through the study

We identified 417 patients with blunt chest trauma of which 369 were included in the study (Fig. 1). Mean age was 56.3 (SD ± 19.5) and 220 (59.6%) were male. 274 patients (74.3%) were discharged home from the ED, and 95 (25.7%) were admitted to hospital from the ED.

Falling to the same level (i.e. from own height) was the most common trauma mechanism (*n* = 199, 53.9%) (Table 2). Most patients had isolated chest trauma (*n* = 319, 86.4%) but some has associated injuries most commonly limb fractures (Table 2). 126 (34.1%) patients had rib fractures with a mean of 1.1 (SD ± 1.9) fractures. CXR was performed in 264 patients (71.5%), CT chest in 87 (23.6%) and CT abdomen in 78 patients (21.1%). In two cases, rib fractures were documented on CT spine. No imaging was available in 92 patients (24.9%), all of whom were discharged with only one patient reattending due to persistent chest pain. 27 (7.3%) patients were on anticoagulants and 30 (8.1%) had a medical history of chronic lung disease. 95 of 369 patients (25.7%) were admitted of whom 53 (14.4%) were admitted to the critical care unit. 274 (74.3%) were discharged from the ED. No patient required tracheal intubation.

**Table 2:** Baseline patient characteristics

Variable^a^	Total (*n* = 369)	Discharged (*n* = 274)	Admitted (*n* = 95)
Age, mean ± SD	56.3 ± 19.5	52 ± 18.1	69 ± 17.6
Sex, *n* (%)			
Female	149 (40.4)	112 (40.9)	37 (38.9)
Male	220 (59.6)	162 (59.1)	58 (61.1)
Injury mechanism, *n* (%)			
Falling to the same level	199 (53.9)	151 (55.1)	48 (50.5)
Falling to a lower level	63 (17.1)	38 (13.9)	25 (26.3)
Direct chest trauma	19 (5.1)	17 (6.2)	2 (2.1)
Assault	21 (5.7)	19 (6.9)	2 (2.1)
Sporting accident	18 (4.9)	18 (6.6)	0
Road traffic accident	47 (12.7)	31 (11.3)	16 (16.8)
Car	20 (5.4)	12 (4.4)	8 (8.4)
Motorbike	6 (1.6)	3 (1.1)	3 (3.2)
Bike	19 (5.1)	15 (5.5)	4 (4.2)
Pedestrian	2 (0.5)	1 (0.4)	1 (1.1)
Unknown mechanism	2 (0.5)	0	2 (2.1)
Isolated chest trauma, *n* (%)	319 (86.4)	260 (94.9)	59 (62.1)
Other injury, *n* (%)	50 (13.6)	14 (5.1)	36 (37.9)
Head	7 (1.9)	0	7 (7.4)
Abdomen	2 (0.5)	0	2 (2.1)
Spinal	13 (3.5)	3 (1.1)	10 (10.5)
Pelvic	5 (1.4)	0	5 (5.3)
Limbs	35 (9.5)	11 (4)	24 (25.3)
Anticoagulation, *n* (%)	27 (7.3)	9 (3.3)	18 (18.9)
Chronic lung disease, *n* (%)	30 (8.1)	11 (4.0)	19 (20.0)
Patients with rib fractures, *n* (%)	126 (34.1)	41 (15.0)	85 (89.5)
Number of rib fractures, mean ± SD	1.1 ± 1.9	1.6 ± 1.5	3.2 ± 2.3
SpO_2_, mean ± SD	96.9 ± 3.1	97.7 ± 1.5	94.6 ± 4.6
95–100, *n* (%)	284 (77.0)	228 (83.2)	56 (58.9)
90–94, *n* (%)	36 (9.8)	7 (2.6)	29 (30.5)
85–89, *n* (%)	5 (1.4)	0	5 (5.3)
80–84, *n* (%)	5 (1.4)	0	5 (5.3)
Unknown, *n* (%)	39 (10.6)	39 (14.2)	0
SpO_2_ on RA, *n* (%)	357 (96.7)	274 (100)	83 (87.4)
SpO_2_ on O_2_, *n* (%)	12 (3.3)	0	12 (12.6)
Sternal fracture, *n* (%)	16 (4.3)	8 (2.9)	8 (8.4)
Flail chest, *n* (%)	9 (2.4)	1 (0.4)	8 (8.4)
First 4 rib fractures, *n* (%)	34 (9.2)	6 (2.2)	28 (29.5)
Respiratory rate, mean ± SD	17.7 ± 3.8	16.8 ± 2.0	20.1 ± 5.1
Comorbidities			
DM, *n* (%)	32 (8.7)	18 (6.6)	14 (14.7)
IHD, *n* (%)	27 (7.3)	16 (5.8)	11 (11.6)
Asthma, *n* (%)	23 (6.2)	18 (6.6)	5 (5.3)
Alcohol dependence, *n* (%)	20 (5.4)	8 (2.9)	12 (12.6)
Psychiatric disorder, *n* (%)	14 (3.8)	11 (4.0)	3 (3.2)
CKD, *n* (%)	12 (3.3)	3 (1.1)	9 (9.5)
Active cancer, *n* (%)	12 (3.3)	5 (1.8)	7 (7.4)
Dementia, *n* (%)	12 (3.3)	2 (0.7)	10 (10.5)
Drug addiction, *n* (%)	10 (2.7)	6 (2.2)	4 (4.2)
Cirrhosis, *n* (%)	4 (1.1)	3 (1.1)	1 (1.1)

^a^*SpO*_*2*_ oxygen saturation level, *RA* room air, *O*_*2*_ oxygen, *DM* diabetes mellitus, *IHD* ischaemic heart disease, *CKD* chronic kidney disease

### Development of complications

62 patients (16.8%) developed complications, 52 of these were patients admitted to hospital and 10 were patients discharged home. Commonest complications were LRTI (*n* = 36, 9.8%) and the presence of consolidation on imaging (*n* = 34, 9.2%). Pneumothorax and haemothorax were present in 18 patients (4.9%) and 9 patients (2.4%), respectively; of these 24 of 27 recovered after conservative treatment and 3 patients needed chest tube insertion. An associated abdominal injury was present in two patients, one had splenic injury and one hepatic injury. No patient developed empyema. There were five deaths in total, all in patients aged > 70 years and all of whom had a score ≥ 16.

### STUMBL score

Mean STUMBL score was 9.3 (SD ± 8.0). The risk score and corresponding risk of developing complications is shown in Table 3.

**Table 3:** Risk score and corresponding risk of developing complications (*n* = 369)

Score	Probability of complications (%)	Number of patients in each category *n* (% of population)
1–10	5.1	252 (68.3%)
11–15	24.5	53 (14.4%)
16–20	53.8	23 (6.2%)
21–25	68.4	19 (5.2%)
26–30	69.2	13 (3.5%)
31+	77.7	9 (2.4%)

In the discharged population, 240 patients (87.6%) had a STUMBL score ≤ 10 and 34 (12.4%) a score ≥ 11, mean score was 6.0 (SD ± 4.0). Most of the 22 patients who reattended the ED did so due to ongoing chest pain but 5 required admission for respiratory failure. These five all had a score ≥ 11, mean score 17.8 (SD ± 10.7), compared to patients discharged again who all (except one patient), had a score ≤ 10, mean score 6.4 (SD ± 4.0).

**Fig. 2:**
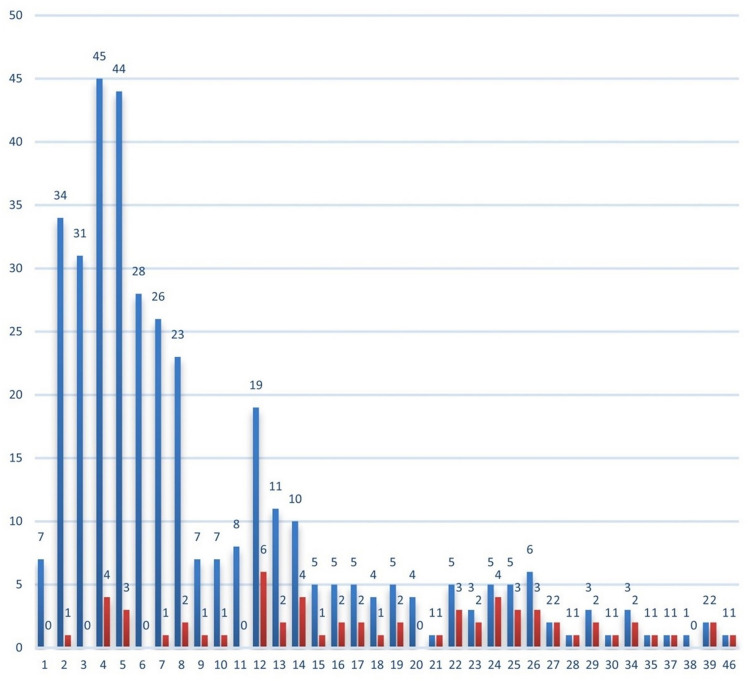
Risk of complications for each STUMBL score (blue: total with score, red: number with complication)

**Fig. 3:**
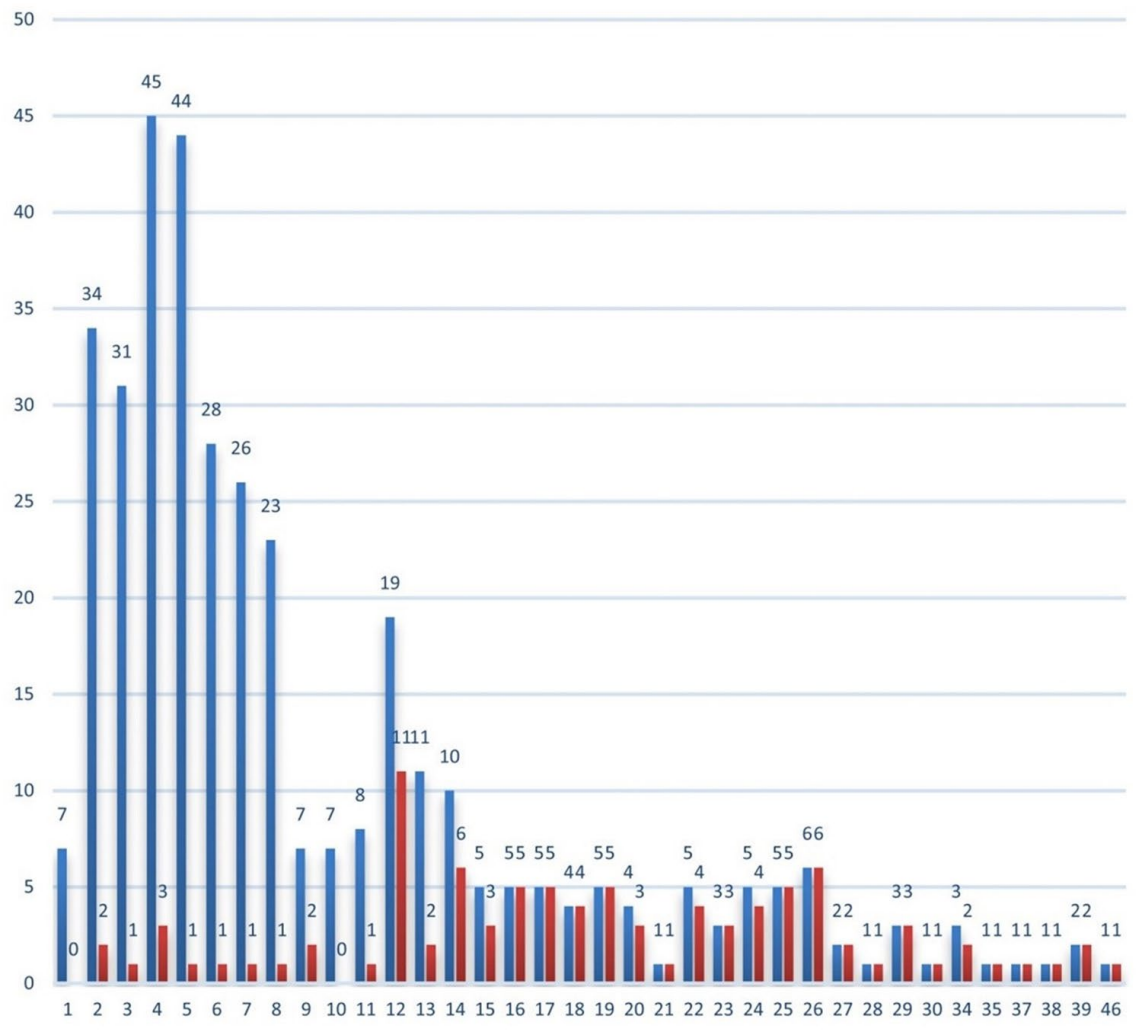
Admission/discharge decision for each STUMBL score (blue: total with score, red: number admitted to hospital)

In the admitted population, 83 patients (87.4%) had a score ≥ 11 and 11 patients (11.6%) a score ≤ 10; mean score was 19.4 (SD ± 8.9). Figure 2 details the risk of complications for each STUMBL score and Fig. 3 details the admission/discharge decision for each STUMBL score.

### Performance of STUMBL score ≥ 11 for predicting complications

Test characteristics for STUMBL score ≥ 11 predicting of complications were: sensitivity = 79.0%, specificity = 77.9%, PPV = 41.9% and NPV = 94.8%. The ROC curve for STUMBL score and risk of complication had an area under the curve (AUC) of 0.84 (95% CI 0.78–0.90).

### Performance of ED clinician decision (decision to admit) for predicting complications

Test characteristics for ED clinician decision to admit for predicting complications were: sensitivity = 83.9%, specificity = 86.0%, PPV = 54.7% and NPV = 96.4%. The ROC curve for ED clinician decision to admit and risk of complication had an AUC of 0.85 (95% CI 0.79–0.91).

ED clinicians admitted 95 of 369 patients, and 52 developed complications. Admitting everyone with a STUMBL score of ≥ 11 would have led to 117 of 369 patients being admitted with only 49 of them developing complications.

### Performance of STUMBL score ≥ 11 for predicting LRTI

Test characteristics for STUMBL score ≥ 11 predicting of LRTI were: sensitivity = 83.8%, specificity = 74.1%, PPV = 26.5% and NPV = 97.6%. The ROC curve for STUMBL score and risk of LRTI complication had an AUC of 0.84 (95% CI 0.78–0.91).

### Performance of ED clinician decision (decision to admit) for predicting LRTI

Test characteristics for ED clinician decision to admit for predicting LRTI were: sensitivity = 83.8%, specificity = 80.7%, PPV = 32.6% and NPV = 97.8%. The ROC curve for ED clinician decision to admit and risk of LRTI complication had an AUC of 0.82 (95% CI 0.75–0.90).

Battle et al. also proposed a score ≥ 26 to select patients requiring critical care admission. In our population, 72% of patients with a score ≥ 26 developed complications compared to the 13% of patients with a score ≤ 25. There were five deaths, four of whom had a score ≥ 26.

### Performance of STUMBL score ≥ 26 for predicting of complications

Test characteristics for a STUMBL score of ≥ 26 for predicting complications were: sensitivity = 25.8%, specificity = 98.0%, PPV = 72.7% and NPV = 86.7%.

### Performance of ED clinician decision (decision to admit to critical care) for predicting complications

Test characteristics for ED clinician decision to admit to critical care for predicting complications were: sensitivity = 53.2%, specificity = 93.5%, PPV = 62.3% and NPV = 90.8%.

## Discussion

In this study looking at the clinical effectiveness of the STUMBL score for the management of blunt chest trauma patients in the ED, we found that a STUMBL score ≥ 11 performs no better than ED clinician judgement decision to admit and leads to more patients being admitted to hospital.

To improve the diagnostic accuracy of clinicians, a score should be superior to that of unstructured clinical judgement alone [13, 14]. They are probably more effective when supporting more inexperienced physicians [15]. In our ED, junior doctors are supervised by senior emergency physicians and this could have influenced our results as clinical judgement may have been superior to other Emergency Departments. More work is needed to evaluate if this tool could be helpful in settings with less senior supervision.

Blunt chest wall trauma management in ED is particularly difficult. Whilst many complications can be detected during the first assessment in ED, there is a frequent onset of respiratory complications (9.8% in our study) which develop later [1, 2, 6–9]. Therefore, a clinical decision tool specifically identifying patients at high risk of developing LRTI would be particularly useful. When we compared clinical judgement to a STUMBL score ≥ 11 for specifically predicting the risk of LRTI, clinical judgement still resulted in an equal or better sensitivity, specificity, PPV and NPV.

Battle et al. also proposed a score ≥ 26 as the cutoff point at which the blunt chest trauma was considered a high enough risk to require critical care admission. In this study STUMBL score ≥ 26 showed better specificity and PPV but lower sensitivity and NPV in predicting complications compared to clinical judgement. Only 22 patients (6.0%) had a score ≥ 26; therefore, these results should be interpreted with caution. It should be also considered that critical care admission criteria differ considerably between countries making extrapolation of this part of the predictive tool harder.

The population selected for this study was different in several aspects compared to the original development and validation cohorts. Unlike Battle et al., we decided to include all patients with blunt chest trauma even in the absence of radiological evidence of rib fractures or pulmonary contusion. This decision was driven by desire to select a population that would represent our clinical practice in the ED. This resulted in a lower number of rib fractures [median 0 (IQR 1) versus median 3 (IQR 3) in the original study development sample and median 1 (IQR 3) in the validation sample] and in a higher oxygen saturation value [median 98 (IQR 3) versus median 95 (IQR 5) in the development sample and median 97 (IQR 5) in the validation sample]. Moreover, chronic lung disease was present in only 8.1% of our population (compared to 56% in STUMBL original development cohort/21% in STUMBL validation cohort) and pre-injury anticoagulant use was present in only 7.3% (43%/20%). The complication rate was also lower in our population (16.8% vs 59%/43%) [1].

The selection of complications also differentiated from Battle et al. study. ICU admission was not considered as a complication in our study as we wished to compare STUMBL score ≥ 26 to clinical judgement in selecting patients requiring critical care admission. Prolonged length of stay (LOS) was also not included since this could have been influenced by other injuries. Minor pleural effusion with no evidence of haemothorax was not included as a complication as it was deemed not serious enough to influence patient management. Finally, we decided to include splenic and hepatic injuries as complications as solid organ injury needs to be considered in the evaluation of patients with injury to the lower chest wall particularly the lower ribs.

### Limitations

There are several limitations that should be considered when interpreting the results of this study. This is a single-centre study; therefore, it may not be representative of other hospital populations. Data were obtained retrospectively through medical chart review; consequently, not all data were available. When oxygen saturation was not reported, it was considered normal whilst when it was available only on oxygen, it was considered as recorded on air room. This might have underestimated or overestimated the STUMBL score. Furthermore, the number of rib fractures could have been underestimated when calculated based on only CXR or when no imaging was performed. We did not link to primary care data to further look for complications that developed after hospital discharge but assumed that any significant complication would have resulted in a return to our ED which is the only ED in our Lothian area that sees trauma patients. Although we excluded patients with other injuries requiring critical care admission, the decision to admit a patient to hospital or critical care may have been affected by other factors that we have not considered here (e.g. social support, other comorbidities).

Finally, although anecdotally STUMBL is not commonly in use in our ED, or formally as part of our ED guidelines, it is not clear how many of our faculty physicians use the STUMBL score to decide on admission/discharge decisions. If ED clinicians were using the score as a whole or in part, many admissions could have been based on the STUMBL decision aide which could have influenced our results.

## Conclusions

A STUMBL score ≥ 11 performs no better than ED clinician judgement decision to admit and leads to more patients being admitted to hospital. Further studies are required before the STUMBL score should be routinely adopted into clinical practice.

## Data Availability

Data are available on request.
